# Ecological, beneficial, and pathogenic functions of the Type 9 Secretion System

**DOI:** 10.1111/1751-7915.14516

**Published:** 2024-06-26

**Authors:** Sofia T. Rocha, Dhara D. Shah, Abhishek Shrivastava

**Affiliations:** ^1^ Biodesign Institute Arizona State University Tempe Arizona USA; ^2^ School of Life Sciences Arizona State University Tempe Arizona USA; ^3^ School of Mathematical and Natural Sciences Arizona State University Glendale Arizona USA

## Abstract

The recently discovered Type 9 Secretion System (T9SS) is present in bacteria of the Fibrobacteres–Bacteroidetes–Chlorobi superphylum, which are key constituents of diverse microbiomes. T9SS is instrumental in the extracellular secretion of over 270,000 proteins, including peptidases, sugar hydrolases, metal ion‐binding proteins, and metalloenzymes. These proteins are essential for the interaction of bacteria with their environment. This mini‐review explores the extensive array of proteins secreted by the T9SS. It highlights the diverse functions of these proteins, emphasizing their roles in pathogenesis, bacterial interactions, host colonization, and the overall health of the ecosystems inhabited by T9SS‐containing bacteria.

## Graphical abstract

This mini review delves into the broad spectrum of proteins secreted by the bacterial type 9 secretion system (T9SS). It focuses on the roles of T9SS‐secreted proteins in pathogenesis, bacterial interactions, host colonization, and the maintenance of ecosystem health.

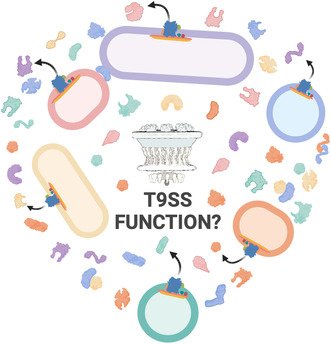

## INTRODUCTION

The Type 9 Secretion System (T9SS) is a complex and specialized secretion machinery found in members of the Fibrobacteres, Bacteroidetes, and Chlorobi (FCB) superphylum. T9SS is remarkable for its role in the secretion of a wide array of proteins that function outside the bacterial cell, facilitating various processes critical for bacterial survival and interaction with their environment. Notably, the T9SS is responsible for the secretion of many proteins, encompassing enzymes that degrade polysaccharides, proteins involved in cell adhesion, gliding motility, biofilm formation, and virulence factors. These secreted proteins enable bacteria to effectively colonize diverse habitats, from soil and sediment to the guts of animals, by breaking down complex molecules, adhering to surfaces, and interacting with host organisms.

The T9SS is intricately designed, featuring several components that extend across the bacterial inner membrane, periplasm, and outer membrane. T9SS functions as a dual‐purpose rotary machinery—not only facilitating the secretion of proteins but also enabling bacterial gliding motility (Sato et al., 2010; Shrivastava et al., 2013). The rotation of T9SS, crucial for both these functions, is driven by the proton motive force (Shrivastava & Berg, 2020). Specifically, the GldL and GldM stator units, which pair in a 5:2 ratio, harness this proton motive force to generate rotational energy (Hennell James et al., 2021).

The periplasmic region of GldM engages with the GldKN ring located at the periplasmic side of the outer membrane. This supports the localization of several SprA translocons on the GldKN ring. For a detailed description of the architecture of T9SS, readers are encouraged to refer to recent reviews and research articles (Paillat et al., 2023; Song et al., 2022; Trivedi et al., 2022). In this review, we focus on summarizing the current knowledge regarding the involvement of T9SS in pathogenicity, immunomodulation, host colonization, facilitation of beneficial host‐bacterial and interbacterial interactions, polysaccharide degradation, environmental adaptation, and the diversity of proteins secreted by T9SS.

## FUNCTIONAL DIVERSITY OF T9SS‐SECRETED PROTEINS

**FIGURE 1:**
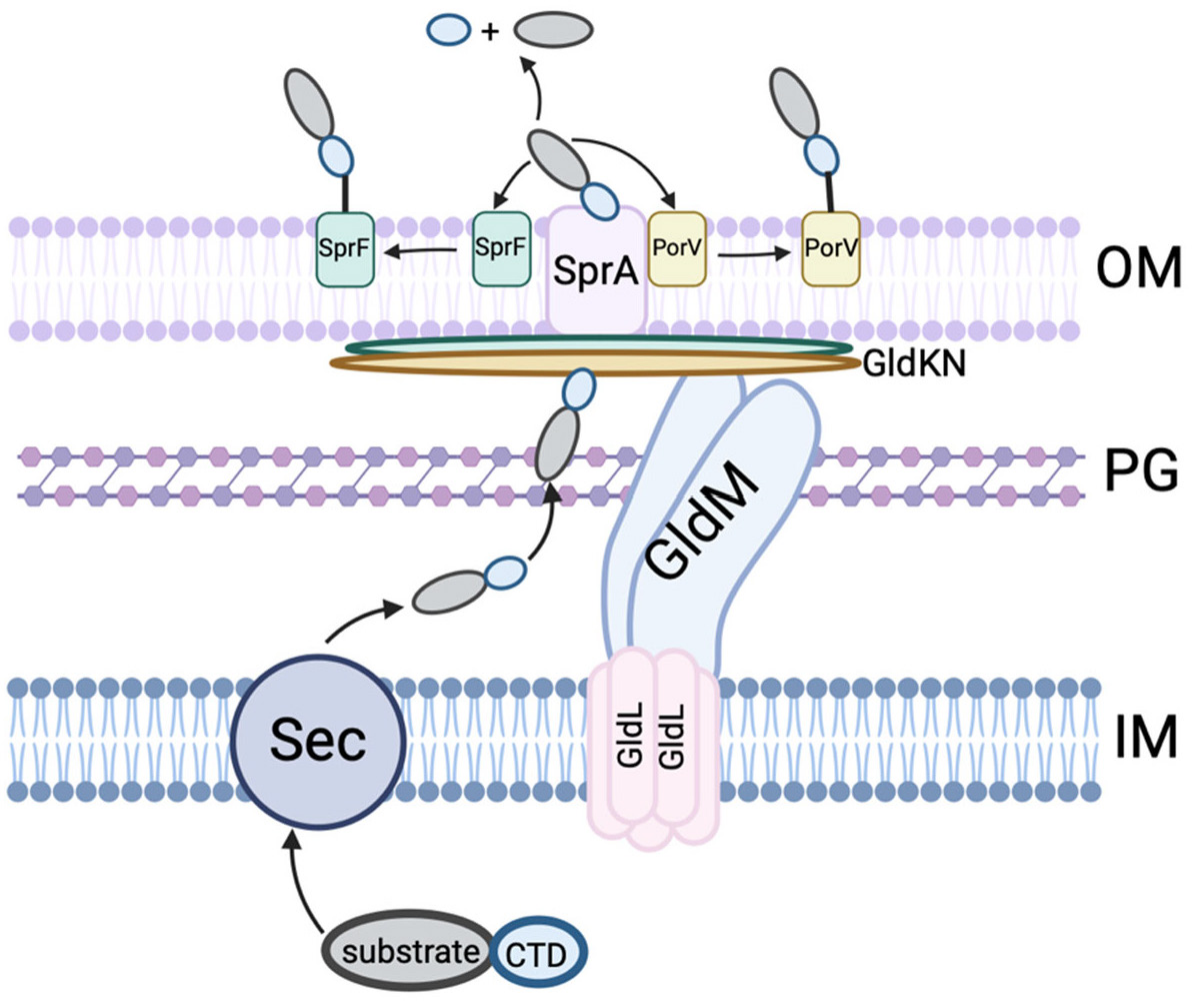
A diagram illustrating the pathways through which proteins are secreted and localized by the T9SS.

T9SS features a unique protein‐sorting mechanism, where proteins destined for secretion are recognized via a C‐terminal domain (CTD) signal and then translocated across the outer membrane through a dedicated channel. There are two types of CTD signals: Type A and Type B. After secretion, proteins with a Type A CTD bind to the outer membrane protein PorV and are shuttled to an attachment complex composed of PorU, PorZ, PorQ, and PorV. In the human oral pathogen *Porphyromonas gingivalis*, the T9SS‐secreted proteins are cleaved by PorU, forming an intermediate acyl‐enzyme with the mature C‐terminus of the CTD protein. A‐LPS are thought to resolve the intermediate and bind CTD proteins to the cell membrane. In contrast, proteins with a Type B CTD bind to an outer membrane beta‐barrel protein, PorP/SprF (Figure 1) (Gorasia et al., 2015, 2022; Kulkarni et al., 2017; Lasica et al., 2017).

**FIGURE 2:**
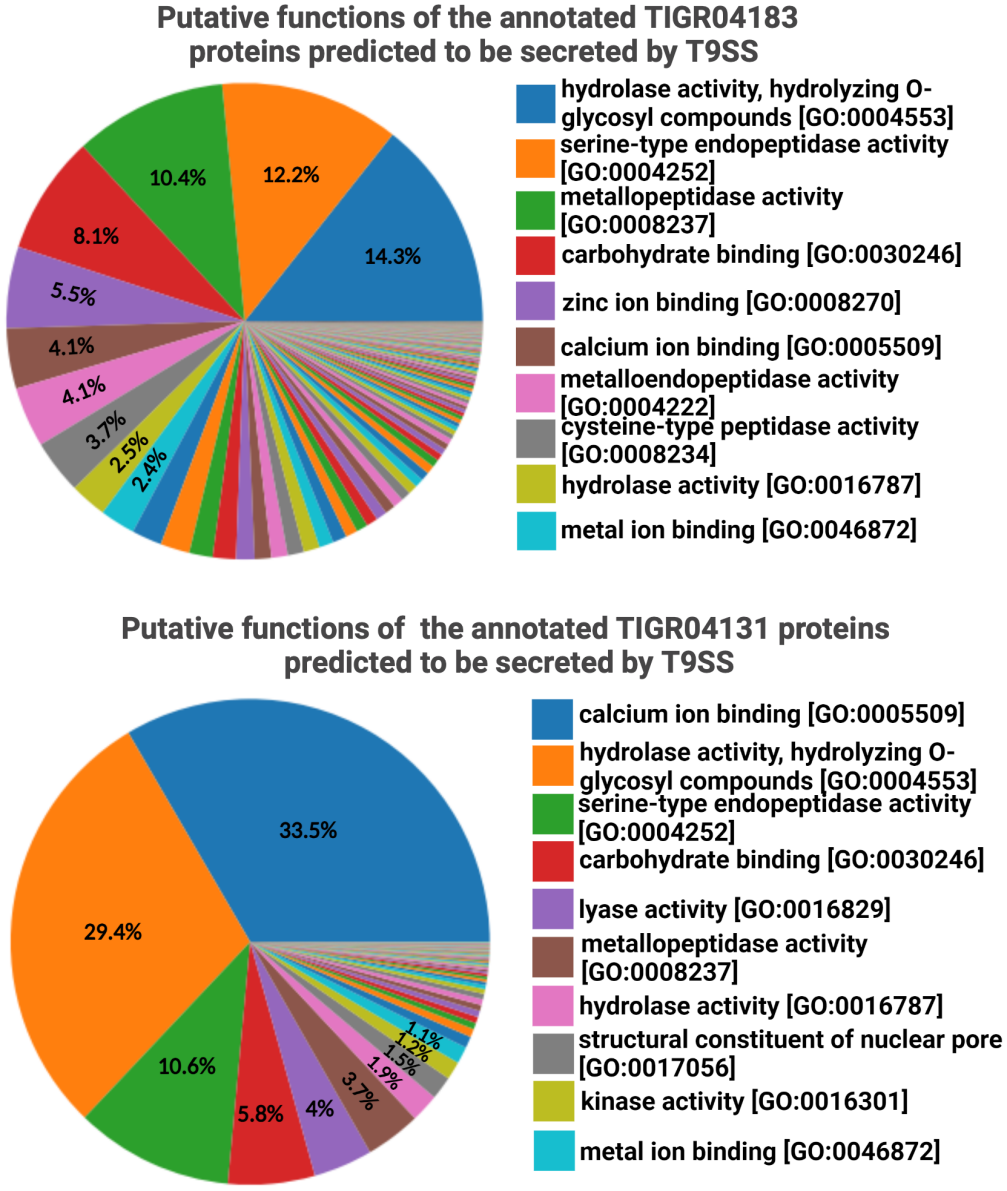
Putative functions and abundance of the annotated bacterial enzymes and proteins predicted to be secreted by Type 9 Secretion System.

Type A and Type B CTDs are categorized as TIGR families TIGR04183 and TIGR04131, respectively (Figure 2). In the UniProt database (The UniProt Consortium, 2023), over 238,000 different proteins are catalogued in TIGR04183, while over 35,000 different proteins are catalogued in TIGR04131 (Figure 2). Around 30% of the 238,000 TIGR04183 containing proteins catalogued on UniProt are annotated by GO terms. Functional classification of these proteins shows that the topmost predicted categories by abundance are glycosyl hydrolases (14.3%, GO:0004553), serine‐type endopeptidases (12.2%, GO:0004252), metallopeptidases (10.4%, GO:0008237), carbohydrate‐binding proteins (8.1%, GO:0030246), zinc ion‐binding proteins (5.5%, GO:0008270), calcium ion‐binding proteins (4.1%, GO:0005509), metalloendopeptidase (4.1%, GO:0004222), and cysteine‐type peptidase (3.7%, GO:0008234). 10% of around 35,000 TIGR04131 containing proteins catalogued on UniProt are annotated by GO terms. The topmost categories by abundance for the functionally annotated TIGR04131 containing proteins are calcium ion‐binding proteins (33.5%, GO:0005509), glycosyl hydrolases (29.4%, GO:0004553), serine‐type endopeptidases (10.6%, GO:0004252), carbohydrate‐binding proteins (5.8%, GO:0030246), lyase activity (4%, GO:0016829), and metallopeptidase (3.7%, GO:0008237). The roles of prevalent protein families secreted by T9SS are detailed in the following summary. Since T9SS is a recently discovered system, knowledge about the metallopeptidases, metal‐binding proteins, and lyases predicted to be secreted by the Type 9 Secretion System (T9SS) is currently limited; hence, a summary of these families is not included in this review.

Cellulose is one of the most abundant organic compounds on Earth and a prime candidate for biofuel production. However, its crystalline structure makes it highly resistant to hydrolysis. The main challenge in degrading cellulose lies in accessing the crystalline cellulose chain due to its structural recalcitrance. Proteins containing carbohydrate‐binding modules, expansins and expansin‐like proteins, and lytic polysaccharide monooxygenases have been identified as key in disrupting the hydrogen‐bonding networks in cellulose. *Cytophaga hutchinsonii*, a widely prevalent cellulolytic bacterium from the Bacteroidetes phylum, demonstrates a unique T9SS‐dependent mechanism for degrading crystalline cellulose. Unlike most cellulolytic bacteria, *C. hutchinsonii* neither secretes a free‐cellulase system nor forms cellulosomes. Recent studies have revealed that T9SS plays a crucial role in the cellulose degradation capabilities of *C. hutchinsonii* by facilitating the secretion of proteins that degrade the crystalline region of cellulose (Wang et al., 2016), and it is vital for cellulose utilization (Gao et al., 2022).

Chitin, a polymer of β‐1,4‐linked *N*‐acetyl‐d‐glucosamine (GlcNAc), is a major component of the exoskeletons of arthropods and cell walls of fungi. T9SS plays a pivotal role in the secretion of chitinase, an enzyme responsible for breaking down chitin into a simpler molecule like *N*‐acetylglucosamine. *Flavobacterium johnsoniae* utilizes T9SS to secrete the chitinase ChiA which is a carbohydrate‐binding glycoside hydrolase primarily detected in the cell‐free spent medium (Kharade & McBride, 2014). The degradation of chitin has significant ecological implications, particularly in soil and aquatic ecosystems where chitin is abundant. It contributes to the recycling of nitrogen and carbon and the maintenance of ecological balance (Beier & Bertilsson, 2013). From a biotechnological perspective, understanding T9SS‐mediated chitin degradation can lead to advances in waste management, bioconversion processes, and the production of valuable chitin‐derived products.

Glycosyl hydrolases that hydrolyse O‐glycosyl compounds are among the most abundant types of proteins secreted by T9SS yet very little is known about their role in T9SS‐containing bacteria. Research from non‐T9SS‐containing microbes shows that glycosyl hydrolases play a role in bacterial colonization and biofilm formation. Mucins, which are highly O‐glycosylated compounds (Arike & Hansson, 2016), are readily broken down by gut Bacteroidetes that thrive in mucin‐rich environment (Glover et al., 2022). It is proposed that mucus plays a role in the selective colonization of commensal bacteria and could serve as a dietary source for some microbes (Martens et al., 2008). A glycosyl hydrolase, nghA from *Yersinia pseudotuberculosis*, is capable of cleaving β‐linked *N*‐acetylglucosamine residues and diminishing *in vitro* biofilm formation by *Yersinia pestis*, which is the causative agent of bacterial plague (Erickson et al., 2008). The PgaB proteins from *Bordetella bronchiseptica* and *Escherichia coli* also exhibit glycoside hydrolase activity. The enzymes effectively degrade deacetylated Poly‐β(1,6)‐*N*‐acetyl‐d‐glucosamine (PNAG) which serves as a crucial biofilm component across various pathogenic bacteria. Degradation of PNAG leads to the disruption of PNAG‐dependent biofilms produced by *Bordetella pertussis*, *Staphylococcus carnosus*, *Staphylococcus epidermidis*, and *E. coli* (Little et al., 2018). Whether T9SS‐secreted glycosyl hydrolases play similar roles in the physiology of FCB bacteria remains to be seen.

T9SS is responsible for the secretion of numerous carbohydrate‐binding proteins, including mobile cell‐surface adhesins. SprB, a large cell‐surface adhesin, plays a crucial role in the surface motility of *F. johnsoniae* on agar. When SprB is absent, the motility of *F. johnsoniae* cells on glass surfaces is greatly reduced, although they are not entirely immotile (Nelson et al., 2008). Additionally, the *F. johnsoniae* genome contains several paralogs of SprB, with RemA being another notable mobile cell‐surface adhesin. RemA possesses a lectin domain that specifically binds to rhamnose and galactose, leading to significantly increased cellular aggregation upon overexpression (Shrivastava et al., 2012). This suggests that the interaction of RemA with polysaccharides promotes microbial community formation by facilitating cell–cell and cell–surface interactions.

T9SS is predicted to secrete multiple types of peptidases, out of which serine‐type endopeptidases are most abundant (Figure 2). Driven by a catalytic triad, which includes a serine molecule acting as a nucleophile, they facilitate the breakdown of internal alpha‐peptide bonds within a polypeptide chain. The serine is activated through a proton relay that involves an acidic residue, such as aspartate or glutamate, and a basic residue, typically a histidine (Ekici et al., 2008). Some pathogenic bacteria attach to host surfaces and release proteases that break down host proteins. In fact, serine‐type endopeptidase activity is over‐represented in gut microbiome of ulcerative colitis patients (Thuy‐Boun et al., 2022). As described later in this review, a serine endopeptidase (subtilisin) secreted by T9SS of the pathogenic *R. anatipestifer* is a putative immunomodulator (Guo et al., 2017).

Cysteine peptidases are abundant in the dataset of proteins predicted to be secreted by T9SS (Figure 2). They catalyze the hydrolysis of peptide bonds through a mechanism in which a cysteine residue at the active site acts as a nucleophile. The virulent gingipains RgpA, RgpB, and Kgp are cysteine peptidases (de Diego et al., 2014), and their impact on host physiology is discussed later in this text. It is known that calcium binds to the mature RgpB enzyme (de Diego et al., 2013). Interestingly, calcium ion‐binding proteins are abundant in the dataset of TIGR04131‐containing proteins predicted to be secreted by T9SS. Several of these calcium ion‐binding proteins are annotated as adhesins and often occur in combination with other Gene Ontology (GO) terms. However, the cellular functions of T9SS‐secreted calcium ion‐binding proteins are largely unknown.

## ROLE OF T9SS IN PATHOGENICITY, IMMUNOMODULATION, AND HOST COLONIZATION

A striking feature of the T9SS is its involvement in the virulence of some pathogens. T9SS of the human oral pathogen *P. gingivalis* is responsible for the secretion of gingipains, which are cysteine proteases that degrade tissue and modulate the immune system (Veith et al., 2017; O'Brien‐Simpson et al., 2003). The proteolytically active gingipains target a broad spectrum of host molecules, including antibacterial peptides, components of the complement system, antibodies, cytokines, and various cell‐surface proteins. The targeted cleavage of protease‐activated receptors (PARs) by gingipains activates pro‐inflammatory responses in a variety of cells, including platelets, gingival epithelial cells, and neutrophils. This leads to gingivitis, which causes symptoms like redness, swelling, and bleeding of the gums. The hijacking and deregulation of host pathways contribute to chronic inflammation, creating an environment in which *P. gingivalis* and other inflammation‐loving pathobionts can thrive (Bryzek et al., 2019; Lourbakos et al., 1998). Additionally, the persistent inflammation and immune evasion tactics employed by *P. gingivalis* facilitate the development of dysbiotic oral biofilms. Within these biofilms, periodontal pathogens proliferate in the anaerobic conditions of periodontal pockets, further exacerbating the disease process. When this condition progresses, it can develop into periodontitis, a more severe form of gum inflammation that causes deterioration of the gums and bones, eventually leading to bone loss.

The impact of T9SS on the human oral microbiota extends beyond oral health. It was recently shown that the gingipains secreted by T9SS exhibit neurotoxic properties, implicating them in neurodegenerative diseases such as Alzheimer's. Gingipains were found in post‐mortem brain samples of Alzheimer's patients, and they correlated with tau and ubiquitin pathology. Infection in mice increased Aβ₁₋₄₂ production, a key component of amyloid plaques. Small‐molecule inhibitors targeting gingipains reduced bacterial load, blocked Aβ₁₋₄₂ production, reduced neuroinflammation, and rescued neurons, suggesting their potential in treating neurodegeneration in Alzheimer's disease (Dominy et al., 2019).

Generation of T9SS mutants of another human oral microbe *Tannerella forsythia* has shed light on its role in the regulation of surface layer (S‐layer), glycoprotein glycosylation, haemagglutination, and biofilm formation. These findings not only demonstrate the involvement of T9SS in the secretion and assembly of virulence factors but also underscore its importance in bacterial adherence and immune evasion strategies. The diminished haemagglutination and altered biofilm dynamics observed in the mutants highlight the diverse role of T9SS in host interaction and colonization (Narita et al., 2014). Another study compared the immune response elicited by wild‐type strains and T9SS‐deficient mutants of *T. forsythia* and *P. gingivalis*. The expression and production of pro‐inflammatory mediators IL‐6, IL‐8, MCP‐1, and TNF‐α were assayed upon bacterial stimulation. The findings revealed a nuanced impact of T9SS on host immune responses, with *T. forsythia* mutants inducing a significantly diminished inflammatory response, whereas *P. gingivalis* mutants elicited an increased response. This differential effect underscores the complexity of T9SS's involvement in periodontal pathogenesis, meriting further exploration to fully understand its role and therapeutic value in managing periodontal diseases (Braun et al., 2022).

It is reported that *Prevotella intermedia*, a Gram‐negative bacterium found in periodontal pockets and linked to various infections, secretes virulent Interpain A, a cysteine protease, via its T9SS. *P. intermedia* mutants lacking T9SS also have defects in pigmentation, haemagglutination, and biofilm formation (Naito et al., 2022). *P. intermedia* often exhibits resistance to the human complement system. This resistance is significantly enhanced by Interpain A. Upon incubation with human serum, Interpain A decreases the ability of the serum to kill bacteria. Clinical observations reveal that strains expressing Interpain A show greater resistance to serum than those lacking it, a phenomenon that is notably reversed in the presence of a cysteine protease inhibitor. The mechanism underlying the protective effect of Interpain A involves the inhibition of all three complement pathways, primarily through the degradation of the C3 alpha chain, a key complement factor. Interpain A also activates the C1 complex, leading to the deposition of C1q on various surfaces, which may facilitate early infection stages by promoting local inflammatory responses advantageous to the pathogen's survival (Potempa et al., 2009). A functional T9SS is essential for the secretion of Interpain A (Naito et al., 2022).

*Prevotella melaninogenica* is commonly found in the microbiome of the human upper respiratory tract. It plays a significant role as a pathogen in a variety of anaerobic infections, frequently occurring alongside both aerobic and anaerobic bacteria. The involvement of *P. melaninogenica* in aspiration pneumonia highlights its clinical relevance; however, there is a notable gap in knowledge concerning its specific virulence factors. Understanding these factors is crucial for elucidating the mechanisms by which *P. melaninogenica* contributes to infections and interacts with other bacteria within mixed microbial communities. It was shown that a *P. melaninogenica* mutant strain lacking the T9SS gene, *porK* exhibits reduced haemagglutination, biofilm formation, and protease secretion. Notably, the *porK* mutant demonstrated lower virulence in mice, indicating the role of T9SS in the pathogenicity of *P. melaninogenica* (Kondo et al., 2018).

*Riemerella anatipestifer* is a key pathogen that causes septicaemia in ducklings. Deleting the T9SS core protein gene, *sprT*, resulted in impaired secretion of subtilisin‐like serine protease and a gelatinase. T9SS mutants had increased susceptibility to complement‐mediated killing which suggests that the subtilisin‐like serine protease secreted by T9SS might act as immunomodulators (Guo et al., 2017). Bacterial subtilisins are known to have diverse functions, including (i) the maturation and transport of a filamentous haemagglutinin (Coutte et al., 2001), (ii) cleaving the complement component C5a, which attracts polymorphonuclear leukocytes such as neutrophils to sites of infection or inflammation (Cheng et al., 2002), and (iii) simultaneously facilitating adhesion to fibronectin of the host cell (Beckmann et al., 2002).

Some members of the Flavobacterium genus are known for their pathogenicity in aquatic environments. The T9SS of the Flavobacterium genus is responsible for secreting a range of proteins, many of which are virulence factors crucial for survival and pathogenicity. Aquatic species such as *Flavobacterium columnare* and *Flavobacterium psychrophilum* use the T9SS to secrete enzymes and toxins that directly impact fish health. These secreted factors contribute to bacterial colonization and biofilm formation on fish tissues, evasion of the host immune response, and infliction of direct damage to fish scales. In diseases such as Columnaris and Bacterial Cold‐Water Disease (BCWD), T9SS plays a vital role in the ability of bacteria to adhere to, invade, and degrade fish tissues, leading to symptoms like lesions, necrosis, and systemic infections. The involvement of T9SS in *Flavobacterium*‐induced fish diseases has significant repercussions for aquaculture. Outbreaks of these diseases can lead to high mortality rates, reduced growth rates in surviving fish, and substantial economic losses. The challenge is compounded by the adaptability of *Flavobacterium* sp. and their ability to survive in diverse environmental conditions, making disease control and prevention more complex. A *F. columnare* strain MS‐FC‐4, recently developed as a genetic model, was pivotal in delineating the role of the T9SS in Columnaris disease. Deletion of T9SS core genes abated pathogenicity and the inactivation of motility genes or the deletion of T9SS‐secreted peptidases and cytolysin reduced the virulence in rainbow trout and zebrafish models (Thunes et al., 2022, 2024).

*Flavobacterium psychrophilum* causes disease and mortality in aquaculture‐reared salmonids and rainbow trout. This pathogen leads to BCWD, presenting symptoms like tail erosion, saddleback lesions, and internal organ infection, with high mortality in young fish. BCWD poses a substantial challenge to sustainable aquaculture. The T9SS of *F. psychrophilum* is required for gliding motility and the secretion of various proteins. Some of these proteins are predicted to function as adhesins, peptidases, collagenases, endonucleases, and glycoside hydrolases. T9SS and gliding motility mutants of *F. psychrophilum* exhibit reduced virulence (Barbier et al., 2020; Pérez‐Pascual et al., 2017), but it is still unknown which of the T9SS‐secreted proteins cause BCWD.

The secretome of T9SS containing *Tenacibaculum maritimum*, a bacterium responsible for the fish disease tenacibaculosis, revealed intricate details about its pathogenicity. An examination of multiple *T. maritimum* strains across different serotypes suggested variability in extracellular proteolytic and lipolytic activities within different strains of the O4 serotype. A specific strain of O4 was observed to generate outer membrane vesicles (OMVs) that contained a high concentration of TonB‐dependent transporters and T9SS proteins. Potential virulence factors such as a sialidase, chondroitinase, sphingomyelinase, ceramidase, and collagenase were secreted by *T. maritimum* but were not found in the OMVs (Escribano et al., 2023). However, due to the lack of genetic tools, the direct involvement of T9SS of *T. maritimum* in pathogenesis and protein secretion is yet to be determined.

## T9SS MEDIATED BENEFICIAL HOST–BACTERIAL AND INTERBACTERIAL INTERACTIONS

Most studies on the T9SS have concentrated on its architecture, roles in disease causation, surface gliding, biofilm formation, and the breakdown of energy‐rich polysaccharides. However, advancements in the cultivation and genetic manipulation of a wide range of species from the Bacteroidetes phylum are beginning to shed light on the functions of T9SS within polymicrobial communities and its advantageous impacts on hosts. T9SS was recently reported to be crucial in modulating the activity of trypsin in the distal intestine, a factor linked to various intestinal pathologies. It is through the T9SS that *Paraprevotella* strains, derived from the fecal microbiome of healthy individuals, exhibit trypsin‐degrading capabilities. Proteins secreted by T9SS enable these bacteria to bind trypsin to their surface, which promotes the autolysis of trypsin and plays a protective role in the gut. The presence of *Paraprevotella clara* helps to safeguard IgA from trypsin degradation, enhancing the effectiveness of oral vaccines against certain pathogens. Moreover, this mechanism is instrumental in preventing lethal infections from viruses like murine hepatitis virus‐2, which rely on trypsin for cell entry. This correlation is further supported by observations in SARS‐CoV‐2 patients, where a reduced severity of symptoms is linked to the presence of trypsin‐degrading genes in the gut microbiome. Therefore, the role of T9SS in facilitating the colonization of trypsin‐degrading commensals highlights its critical contribution towards maintaining intestinal homeostasis and protecting against pathogen infections (Li et al., 2022).

As reviewed earlier, the presence of T9SS‐containing bacteria in the human oral microbiota is mostly attributed to pathogenesis. However, T9SS is found in non‐pathogenic gliding bacteria of *Capnocytophaga* genus which are abundant in the human oral microbiota (Rocha et al., 2023). If one scales up a T9SS‐driven gliding cell of *Capnocytophaga gingivalis*, it will be like a surface‐dwelling drone with about 30 to 40 moving adhesins that are randomly positioned over the body of the drone. These adhesins, which are covered with a very strong glue, are about a third of the width of the drone, and they stick out normally to the body of the drone. As this drone moves through a dense area where other drones are stuck in traffic or are simply immobile, the appendages stick onto other immobile drones and tow them along. This effectively clears the traffic and creates a new path while also transporting the immobile drones. A bio‐inspired drone of this kind can be utilized to address real‐world challenges humans may face with the growing reliance on autonomous drones. However, evolutionary pressures have already led to the selection of this design to solve problems that microbes encounter in crowded conditions (Shrivastava et al., 2018).

Single cells of *C. gingivalis* are broad‐range transporters, and it has been shown that they carry other non‐motile human oral bacteria of the genera *Prevotella*, *Fusobacterium*, *Streptococcus*, *Actinomyces*, *Parvimonas*, and *Veilonella* as cargo. Swarms of *C. gingivalis* arrange these non‐motile microbes in islands that are surrounded by streams of motile *C. gingivalis* (Shrivastava et al., 2018). T9SS is also required for gliding motility and biofilm formation by a *Capnocytophaga ochracae* (Kita et al., 2016). The human oral microbiota has a well‐defined spatial structure (Mark Welch et al., 2016). It appears that localization of non‐motile microbes due to cargo transportation might seed some of the steady‐state spatial structures observed in the human oral microbiota.

*Flavobacterium johnsoniae*, another model organism to study T9SS‐driven gliding motility, is isolated from soybean rhizosphere (Peterson et al., 2006). It uses gliding motility to swarm over an agar surface (Wolkin & Pate, 1984). A recent investigation has further detailed this behaviour, revealing that at high densities, the cells interconnect and navigate in counterclockwise paths when subjected to nutrient‐deprived conditions (Nakane et al., 2021). In an under‐oil open microfluidic system, *F. johnsoniae* forms surface‐associated, biofilm‐like microcolonies that demonstrate formation, movement, merging, and dispersion. It was found that the movement of these microcolonies is due to the gliding cells at the base, which attach to the surface at one cell pole. For a single cell to glide, the cell body aligns horizontally with a surface. However, in the microcolony, base cells in contact with the surface do not align horizontally but rather at an oblique angle at one of their poles. The base cell then attaches with the rest of the microcolony via the motile cell‐surface adhesins, and it drives the motility of this self‐assembled biofilm‐like microcolony (Li et al., 2021). In the soybean root microbiota, *F. johnsoniae* exhibits a complex relationship with non‐T9SS‐containing *Bacillus cereus* and *Pseudomonas koreensis*. Specifically, peptidoglycan from *B. cereus* was found to promote the growth of *F. johnsoniae* in root exudate medium. Additionally, Cytophaga‐Flavobacterium bacteria were observed to secrete compounds that degrade *B. cereus* cell walls, indicating a complex interaction between these bacterial groups in the plant rhizosphere (Lozano et al., 2019; Peterson et al., 2006).

Some of the examples discussed thus far demonstrate how gliding motility shapes the structure of a biofilm. In contrast, T9SS‐driven gliding motility can also break the ‘shield’ of biofilms thus making them susceptible to phage infection. Using fluorescently labelled lambda phages, which do not infect *C. gingivalis*, it was discovered that these phages were actively transported by *C. gingivalis* towards an *E. coli* colony, enhancing the rate of phages into the *E. coli* colony by tenfold as compared to passive diffusion. Moreover, *C. gingivalis* swarms were able to create tunnel‐like structures in *E. coli* biofilms, facilitating deeper phage penetration. This suggests that *C. gingivalis* not only alters the physical structure of its target biofilms but also significantly increases the efficacy of phage invasion, revealing a complex interaction mechanism within the oral microbiota that may impact microbial community dynamics and pathogen transmission (Ratheesh et al., 2023).

Recent evidence suggests a role for T9SS in algal bacterial interactions. In a co‐culture experiment, a T9SS‐containing bacterium *Dyadobacter* sp. HH091 attached to the microalgae *Micrasterias radians*. Transcriptome analysis pointed towards an elevation in the expression of T9SS genes that could secrete factors for adhesion that could promote a mutually beneficial relationship. Here, the bacterium might aid in polysaccharide digestion, potentially benefiting the microalga by providing growth‐promoting substances, and in turn, the bacterium benefits from the degradation of algal polysaccharides (Astafyeva et al., 2022). Another recent study on the predatory behaviour known as ixotrophy involves the T9SS. Here, the initiation of predator–prey interaction is facilitated by gliding motility and the use of extracellular proteins resembling grappling hooks, which attach to the flagella of the prey. These hooks have been identified through cryo‐electron microscopy as a heptameric structure formed by a protein secreted by the T9SS. After attachment, the predatory bacteria employ the Type 6 Secretion System (T6SS) to puncture and kill prey cells, with the T9SS working in conjunction with T6SS to establish bacterial predatory behaviour (Lien et al., 2024).

## CONCLUDING REMARKS

**FIGURE 3:**
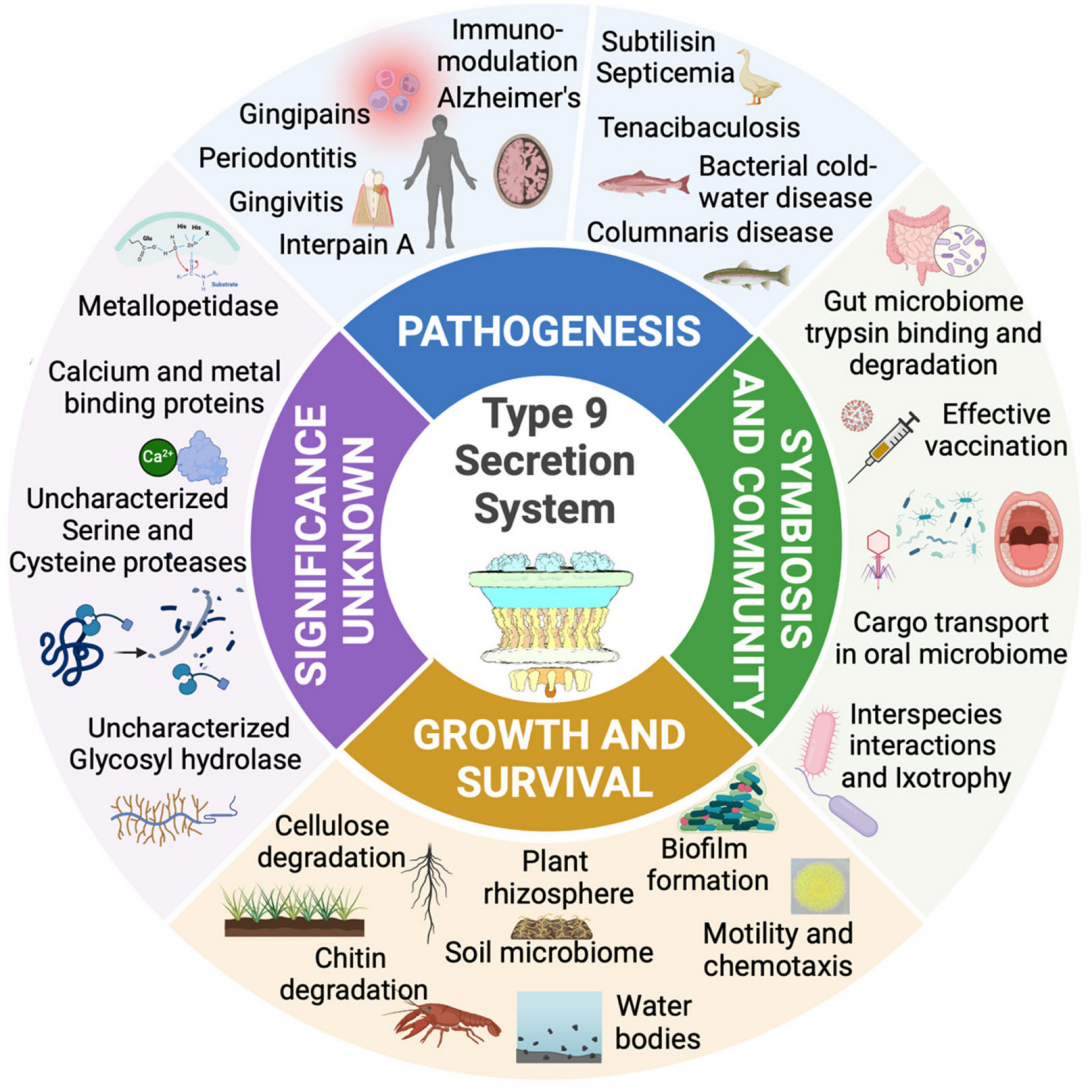
A diagram depicting the diverse functions of the Type 9 Secretion System.

The T9SS plays significant roles in bacterial pathogenesis, colonization of hosts, interbacterial interactions, and the maintenance of ecosystem health (Figure 3). The release of virulence factors through T9SS enhances the ability of bacteria to penetrate host defences, avoid immune detection, and initiate infections. T9SS plays a key role in orchestrating bacterial relationships by altering their habitats, impacting competitive behaviours, and promoting symbiotic associations. Delving into the mechanics and control of T9SS illuminates the survival tactics bacteria deploy in diverse settings, while also highlighting potential targets for novel antimicrobial therapies. Given the staggeringly large number of proteins secreted by T9SS across numerous, yet‐to‐be‐fully‐explored FCB bacteria, it becomes evident that our current understanding of the biological importance of T9SS is just beginning to unfold.

## AUTHOR CONTRIBUTION

STR, DDS, and AS conceptualized and wrote the article.

## CONFLICT OF INTEREST STATEMENT

The authors declare no conflict of interests.
